# MugenNet: A Novel Combined Convolution Neural Network and Transformer Network with Application in Colonic Polyp Image Segmentation

**DOI:** 10.3390/s24237473

**Published:** 2024-11-23

**Authors:** Chen Peng, Zhiqin Qian, Kunyu Wang, Lanzhu Zhang, Qi Luo, Zhuming Bi, Wenjun Zhang

**Affiliations:** 1 School of Mechanical and Power Engineering, East China University of Science and Technology, Shanghai 200237, China; ceciliapengchen@163.com (C.P.); x849545512@hotmail.com (K.W.); lzzhang@ecust.edu.cn (L.Z.); luoqi_ecust@163.com (Q.L.); 2 Department of Engineering, Purdue University, West Lafayette, IN 47907, USA; biz@pfw.edu; 3 Department of Mechanical Engineering, University of Saskatchewan, Saskatoon, SK S7N 5A9, Canada

**Keywords:** transformer, convolutional neural network, polyp detection, image segmentation

## Abstract

Accurate polyp image segmentation is of great significance, because it can help in the detection of polyps. Convolutional neural network (CNN) is a common automatic segmentation method, but its main disadvantage is the long training time. Transformer is another method that can be adapted to the automatic segmentation method by employing a self-attention mechanism, which essentially assigns different importance weights to each piece of information, thus achieving high computational efficiency during segmentation. However, a potential drawback with Transformer is the risk of information loss. The study reported in this paper employed the well-known hybridization principle to propose a method to combine CNN and Transformer to retain the strengths of both. Specifically, this study applied this method to the early detection of colonic polyps and to implement a model called MugenNet for colonic polyp image segmentation. We conducted a comprehensive experiment to compare MugenNet with other CNN models on five publicly available datasets. An ablation experiment on MugenNet was conducted as well. The experimental results showed that MugenNet can achieve a mean Dice of 0.714 on the ETIS dataset, which is the optimal performance on this dataset compared to other models, with an inference speed of 56 FPS. The overall outcome of this study is a method to optimally combine two methods of machine learning which are complementary to each other.

## 1. Introduction

Colonoscopy is an effective technique for detecting colorectal polyps, which are polypoid lesions on the surface of the colonic mucosa within the intestinal lumen. Colorectal polyps commonly lead to intestinal bleeding and inflammatory responses, and in severe cases, they may progress to colon cancers. Colonoscopy is the most effective way to prevent colon cancers, but the procedure may increase the risk of perforation and cause severe discomfort to the patient [1,2,3]. The detection of abnormal polyps is carried out by means of image processing in colonoscopy.

Previous research indicated that the clinical miss rate for colonic polyps can be as high as 25% [4]. The segmentation of polyps from colonoscopy images is the first step in the early detection of colon cancers. Polyp segmentation can be accomplished using graph-based methods (e.g., Grabcut [5], Graphcut [6]), which model an image as a graph structure and employ graph cut operations to separate foreground and background regions. These methods rely on pixel similarity, calculating connection weights between pixels based on low-level features such as color and brightness. Typically, these methods require user intervention, (such as selecting a region or initial labeling) to help establish foreground and background regions for segmentation. While these methods perform well on small images, their computational efficiency decreases as the image resolution increases, limiting their suitability for real-time processing. In addition, due to the variation in size, color, and texture among polyps of similar types, and the unclear boundaries between polyps and the surrounding mucosa, polyp segmentation remains a challenging task. The accuracy and efficiency of polyp segmentation can be improved by deep learning methods [7].

The basic idea behind the study reported in this paper is to combine or fuse Transformer and CNN due to their nearly complementary ability, according to the hybridization principle [8]. The study reported in this paper proposed a new neural network coined MugenNet. Comprehensive experiments were conducted with the MugenNet on five public colon polyp datasets using six performance indicators or indexes. The study also compared MugenNet with other ten neural networks. These models are listed in Section 3. The main contributions of our work are summarized as follows. (1) A novel deep network is proposed for real-time, accurate polyp segmentation. By combining the CNN and Transformer architectures, the network leverages the CNN’s capacity for capturing local information alongside the Transformer’s strength in processing global context, significantly improving segmentation accuracy and speed. (2) We designed a fusion module called the Mugen module, which can dynamically adjust importance weights for each channel. Experiments demonstrate that this module effectively integrates global features while focusing on pixel-level edge features, which is particularly crucial for accurate polyp segmentation. (3) On five public colon polyp datasets, MugenNet achieves an outstanding performance in comparison with the traditional polyp segmentation methods in terms of training and processing speed, making MugenNet suitable for real-time clinical video data streaming applications.

The remaining part of the paper is organized as follows. Section 2 discusses the idea, method, and their implementation into the model MugenNet. Section 3 presents the experiment to validate MugenNet. Section 4 details the conclusion, along with the discussion of some future works.

## 2. Related Work

### 2.1. CNNs for Image Segmentation

Convolutional neural network (CNN) is a well-known image segmentation method that extracts features from pixels or raw information blocks using convolutional operators [9]. U-Net is a classic model for medical image segmentation, with its encoder–decoder structure effectively capturing multi-scale features, making it suitable for a wide range of medical applications [10]. Numerous researchers have proposed improvements upon the U-Net architecture, leading to variants such as ResU-Net [11], EGE-UNet [12], and U-Net v2 [13], which offer enhanced feature extraction capabilities and optimized information transmission. However, the shortcomings of CNN include (1) computational overhead in training of CNN, (2) not effectiveness in dealing with the biomedical images with low resolution, and (3) potential to be over-fitting [14].

### 2.2. Self-Attention Mechanism/Transformers

Transformer is a machine learning process, which takes a series of symbols as the input, and produces the semantics from this series as the output [15]. Transformer is based on the self-attention mechanism. The self-attention mechanism refers to placing more attention on the relevance of a chunk of information. Suppose that there are three pieces of information, A, B, C. If A is closer to B than to C, a higher attention would be given to B than to C (with respect to A). The Vision Transformer (ViT) represents a significant advancement in the application of Transformer architectures to computer vision tasks [16]. In segmentation tasks, ViT can be combined with upsampling or decoding modules to model dependencies between regions, producing more refined segmentation results.

### 2.3. Transformers to Complement CNNs

To a 2D image, CNN does not have different attention values in the scanning process of the chunks of information [17]. This feature of CNN has two disadvantages compared to Transformer: (1) the computational cost with Transformer will be much lower than that with CNN [18], because CNN must have a considerable amount of redundant computations; (2) Transformer is free of the overfitting problem, suggesting that it be more accurate than CNN. However, one shortcoming of the Transformer/self-attention mechanism is that the approach is highly dependent on the assessment of the relevance, which is further specific to different applications. For instance, in the database called ImageNet [19], such an assessment must rely on external datasets, which is in essence a kind of pre-training process. This shortcoming of Transformer contrasts with the merit of CNN (i.e., the equity among all chunks of information). Overall, CNN-based models [20,21] are unable to capture the global contextual information but are able to capture local information well. However, Transformer-based models [22,23] behave vice versa. Therefore, combining CNN and Transformer is a promising idea.

Currently, the research on effectively combining CNN and Transformer models is still limited. For instance, TransUNet [24] accomplished the combination of CNN and Transformer by employing a chain-like architecture. The method is well-suited for organ recognition tasks because organ boundaries are relatively clear, and uncertainty levels are relatively low. For the definition of uncertainty, we refer to [25]. The system also cannot perform parallel processing, which is one of the important merits of the Transformer. Swin Transformer introduced a multi-scale feature extraction mechanism with hierarchical windows, establishing local attention within each window and using cross-window connections [26]. This approach addressed the high computational cost issue found in ViT. Although the model facilitates information propagation through cross-window connections, the capture of long-range dependencies remains limited.

## 3. The Proposed MugenNet Model

**Figure 1:**
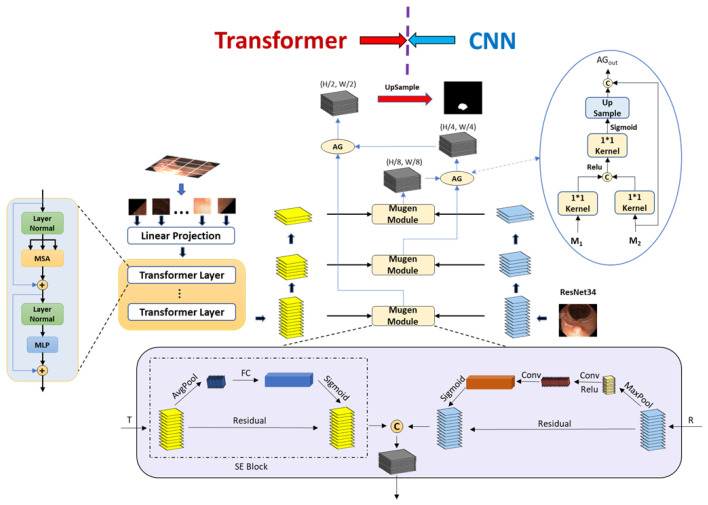
The architecture of MugenNet: combine the Transformer branch (**left**) with the CNN branch (**right**) in the Mugen module (**middle**). FC: Fully Connected. AG: attention gate. SE: Squeeze and Excitation.

MugenNet is mainly composed of a CNN decoder based on Resnet-34 and a Transformer decoder based on ViT, two of which are run in parallel, as shown in Figure 1. The following sections will discuss MugenNet in detail.

### 3.1. Design of the CNN Branch

ResNet-34 [27] was chosen as the CNN branch, as a trade-off between retaining and losing more local information during training. The proposed model used two up-sampling operations to generate three matrices, which stored the feature maps of different scales; as depicted in the inset in the upper right part of Figure 1. These feature maps can be utilized to progressively restore the spatial resolution in subsequent processes. The matrix obtained through the convolution operation in the CNN branch (the right side of Figure 1 served as the input to the Mugen module and fused with the result obtained from the Transformer branch, which will be discussed in the next section.

### 3.2. Design of the Transformer Branch

A pre-trained Vision Transformer (Vit-B/16) was used as the Transformer branch in our model. It is a deep learning model that segments images by dividing them into patches and using self-attention mechanisms to capture global relationships [16]. The input image was divided into 16 blocks, and a self-attention operation was performed on each block. We used two up-sampling operations to generate feature maps, matching their dimensions with the results obtained from the CNN branch. Assuming the input image is $x\in R^{H\times W\times C}$, where H and W represent the length and the width of the original image, respectively, and C is the channel of the image (if it is a color image, C=3, otherwise C=1). First, divide *x* into 4×4 patches, and every patch is $x_{p}\in R^{N\times (P^{2}\times C)}$, where P is the side length of $x_{p}$, (P,P) is the resolution of each patch, and $N=HW/P^{2}$, indicating the number of patches. Then, add a location code to every patch and put each patch into a sequence as the Transformer modules (see the left part of Figure 1). Therefore, each Transformer module consisted of layer normalization (LN), multi-head self-attention (MSA) and multi-layer perceptron (MLP), as depicted in the left part of Figure 1. The use of LN instead of batch normalization was because LN helped us to increase the stability during training with a high learning rate [18,28]. MSA was used to calculate the relevant information learned in different subspaces. MLP was a stack of activation functions and served as the output layer of CNN.

The activation function used is the so-called softmax, which was used to compute the three feature maps (Q, K and V), i.e.,

(1) $$N_{out}=softmax\left(\frac{q_{i}k^{T}}{\sqrt{D_{h}}}\right)v$$

In Equation (1), $N_{out}$ represents the output of each attention module, which was then input into the Mugen module. For the post-processing process, we refer to [29]. Specifically, two consecutive up-sampling convolution layers were connected to the output layer of the Transformer branch to recover the spatial resolution. The feature maps of different dimensions were preserved and fused with the output of the CNN branch.

### 3.3. Mugen Module

The Mugen module was used to combine the global chunks of information from Transformer and CNN, respectively. The process of the combination was composed of two parts. The first part was the Squeeze and Excitation (SE) block [29], and the second part was the module that was built based on the channel attention. The SE block can recalibrate the corresponding characteristics of the channels by explicitly modeling the inter-dependence between the channels and can improve the performance of the neural network without an increase in the computational overhead. The channel attention mechanism learns the weights of importance for each channel, allowing it to dynamically adjust the feature responses of each channel. This enables the network to effectively capture and utilize information from different channels. Such a mechanism aids in improving the performance of the network for specific tasks, and has shown significant improvements in various visual tasks, including image classification, object detection, and semantic segmentation.

In the CNN branch, the channel attention module was adopted [30]. After global max-pooling, the feature map was input into the neural network consisting of two hidden layers. The number of neurons in the first layer was C/r and the number of neurons in the second layer was *C*, where *C* was the number of channels and *r* was the reduction rate. The proposed model removed the global average-pooling part from the Convolutional Block Attention Module (CBAM) to focus more on the pixel-level edge information in the image segmentation task. Finally, the sigmoid function was used to generate the channel attention features.

After the above treatment, two feature maps were obtained: one from the Transformer branch and the other from the CNN branch, respectively. Inspired by ResNet, we added a residual connection architecture (Figure 1) before the final output from the Mugen module. ResNet helps to alleviate the vanishing or exploding gradient problem during the training process.

The steps in the Mugen module include the following computations:

(2) $$\left\{\begin{array}{c} {\hat{t}}^{i}=SeAtten\left(t^{i}\right)\\ {\hat{r}}^{i}=ChannelAtten\left(r^{i}\right)\\ {z^{i}}_{out}=Residual\left(\left[{\hat{t}}^{i},{\hat{r}}^{i},t^{i},r^{i}\right]\right) \end{array}\right.$$

After the Mugen module, a stack of hidden layers with convolution and batch normalization was used to reduce the output dimension to one. The characteristic graph was used as the intermediate output ${z^{i}}_{out}$. We used the progressive up-sampling method to process the characteristic graph obtained from the Transformer and CNN branches.

As shown in Figure 1, initially there were $t^{0},\;r^{0}\in R^{\frac{H}{16}*\frac{W}{16}*D_{0}}$. After two consecutive up-sampling operations, we obtained $t^{1},\;r^{1}\in R^{\frac{H}{8}*\frac{W}{8}*D_{1}}$ and $t^{2},\;r^{2}\in R^{\frac{H}{4}*\frac{W}{4}*D_{2}}$, which restored the spatial resolution to the same dimension as the input image. Finally, we input $t^{i},\;r^{i}$ into the three Mugen modules, respectively, perform feature fusion, and obtain three feature maps, forming the semantic segmentation map.

### 3.4. Details of the Transfer and Loss Function

In the Mugen module, we adopted an architecture to combine Transformer and CNN, called Feature Pyramid (FP), along with the residual, by refining the one proposed by [31]. After the top-down aggregation, the residual architecture was used to gather strong features, which was expected to improve the accuracy of the model. It is noted that compared with other bi-directional methods, the residual FP can more effectively achieve high-precision detection of targets.

From the Mugen module, three feature maps of different dimensions can be obtained. Then, attention gate (AG) was used to restore the resolution of attention. It is worth noting that, according to [32], AG can focus on target architectures of different shapes and sizes, suppress irrelevant regions in the input image, and highlight the target features of specific tasks [33]. The architecture of AG in the Mugen is shown in Figure 1.

**Figure 2:**
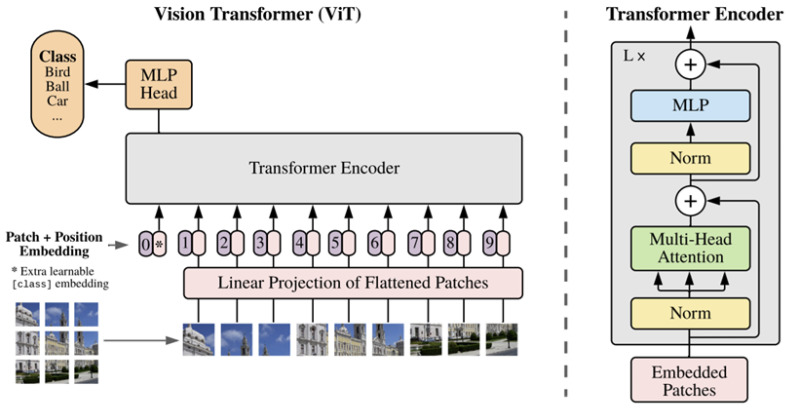
The architecture of the Vision Transformer [16].

From Figure 2, it can be seen that we used two up-sampling operations to gradually restore the original resolution of the feature map. First, we set the output of the first Mugen module $\left({y^{1}}_{out}\right)$ equal to ${z^{1}}_{out}\in R^{\frac{H}{8}*\frac{W}{8}*D_{1}}$. Then, ${z^{1}}_{out}$ and the output of the second Mugen module $\left({y^{2}}_{out}\right)$ were put into the first attention gate to obtain ${z^{2}}_{out}\in R^{\frac{H}{4}*\frac{W}{4}*D_{2}}$. After that, we put ${z^{2}}_{out}$ and the output of the third Mugen module $\left({y^{3}}_{out}\right)$ into the second attention gate to obtain ${z^{3}}_{out}\in R^{\frac{H}{2}*\frac{W}{2}*D_{3}}$. Finally, a separate up-sampling of ${z^{3}}_{out}$ was conducted to obtain the final segmentation graph $z_{out}\in R^{H*W*D}$.

The loss function was defined as $L=L_{IoU}^{\omega }+n*L_{BCE}^{\omega }$ during the training process, according to [32,34], where $L_{IoU}^{\omega }$ was the global weighted average IoU loss (added to the global constraint) and $L_{BCE}^{\omega }$ was the weighted binary cross entropy loss (added to the pixel level constraint). Further, *n* was a hyperparameter used to allocate the proportion of the IoU loss and the BCE loss in the total loss *L*. Compared with the standard IoU loss, which is commonly used in the segmentation tasks, the global average IoU loss highlights its the importance of complex sample pixels by increasing the their weight of the complex sample pixels. At the same time, the weighted binary cross entropy loss pays more attention to the complex sample pixels, which can effectively extract the pixel-level edge features at the pixel level. Considering that the goal of the model was of the colonic polyp segmentation and the edge features were not obvious, we took set n to be 6/5 to increase the proportion of the pixel-level constraints in the overall total loss function. $S_{x}^{up}$ was up-sampled to the same size as the ground-truth graph image *G*. The proposed overall-loss function of MugenNet for colonic polyp detection can then be expressed by:

(3) $$L_{total}=\alpha L\left(G,S_{t}^{up}\right)+\beta L\left(G,S_{r}^{up}\right)+\gamma L\left(G,S_{z}^{up}\right)$$

where $S_{t}^{up}$ and $S_{r}^{up}$ represent the characteristic graph obtained from the Transformer branch and CNN branch, respectively. $S_{z}^{up}$ represents the output of the semantic segmentation graph after up-sampling. The parameters α,β,γ are adjustable hyperparameters.

### 3.5. Implementation

We implemented the MugenNet module (referred to as the model hereafter) in the PyTorch framework on an NVIDIA RTX 3080 GPU. The resolution of input images was shaped to 256×192. The dataset was divided into training, validation, and test sets in the ratio of 7:2:1. The Adam optimizer was used to adjust the overall parameters, with a learning rate of $1\times 10^{-4}$. The batch size was set to 16. The final prediction result was generated after the sigmoid operation.

## 4. Experiment

We compared the MugenNet model with some existing CNN models, which belong to the category of CNN models in terms of abilities such as learning, generalization, and qualitative segmentation.

### 4.1. The Dataset and Model

The experiment was conducted on five publicly available polyp segmentation datasets, namely CVC-300 [35], CVC-ClinicDB [36], CVC-ColonDB [34], ETIS [37], and Kvasir [38]. These datasets contain precisely annotated endoscopic images, covering various resolutions, polyp sizes, and complexities, making them well-suited for polyp detection, segmentation, and model performance evaluation in colorectal imaging. The CVC-300, CVC-ClinicDB, CVC-ColonDB, ETIS, and Kvasir datasets contain 300, 612, 380, 196, and 1000 images, respectively. The first four datasets consist entirely of polyp images, while the Kvasir dataset includes 500 images with polyps and 500 images without polyps. We randomly selected 1450 images from Kvasir and CVC-ClinicDB to train our model, with the weights initialized based on the training weights of DeiT-small [39] and resnet-34 [27]. We then compared our model with ten other convolutional neural network models for biomedical image segmentation, namely U-Net [10], U-Net++ [40], ResUNet++ [41], SFA [42], PraNet [43], DCRNet [44], EU-Net [9], SANet [45], ACSNet [9], and HaeDNet [46].

### 4.2. Training Setting and Performance Indicators

To assess the generalization ability of the model, besides the two datasets used for training the model, we also tested the model on three additional datasets, namely CVC-300, CVC-ColonDB, and ETIS.

### 4.3. Performance Indicators

To evaluate the proposed MugenNet for the segmentation of colon polyp images, we used the performance indicators of mIoU, mDice, MAE loss, $F_{\beta }^{\omega }$, $S_{\alpha }$, and $E_{\xi }$. The mathematical expression for the definition of each performance indicator along with its significance in the image segmentation task is introduced below.

The full name of IoU is Intersection over Union, which represents the ratio of intersection and union between the bounding box and ground truth. The average IoU (mIoU) can be calculated by

(4) $$mIoU=\frac{1}{n}\sum_{i=0}^{n}\frac{A_{i}\cap B_{i}}{A_{i}\cup B_{i}}$$

It is noted that during the training process of a segmentation model, the value of the IoU loss will be used to evaluate whether the prediction result of the model is good or valid. If IoU>0.5, the prediction is considered valid. Additionally, we also calculated the IoU value relative to the ground truth for each training batch, and we then took the average as the mIoU loss for this specific training batch.

The Dice coefficient is a measure of similarity between two sets (for example, A and B) and is used for medical image segmentation. The formula for the mean Dice coefficient (mDice) is as follows:

(5) $$mDice=\frac{1}{n}\sum_{i=0}^{n}\frac{2\left|A_{i}\cap B_{i}\right|}{\left|A_{i}\right|+\left|B_{i}\right|}$$

The range of the Dice coefficient is (0, 1). The closer the coefficient is to 1, the higher the similarity between sets A and B. We calculated the Dice coefficient between the model’s predicted results and the ground truth to evaluate the reliability of the model in colon polyp image segmentation.

The full name of MAE is the Mean Absolute Error, which is used to calculate the error between predicted values and the ground truth. MAE is calculated by

(6) $$MAE=\frac{1}{n}\sum_{i=0}^{n}\left|A_{i}-B_{i}\right|$$

The advantage of MAE is that it is insensitive to outliers. This study took it as one of the criteria to evaluate the reliability of prediction results.

The parameters for the precision and recall metrics include: TP (True Positive), TN (True Negative), FP (False Positive), and FN (False Negative). Each pixel was compared in the map of the prediction result with the ground truth and the prediction result was classified into (i) correct $\left(D\left(i\right)=G\left(i\right)\right)$ or (ii) incorrect $\left(D\left(i\right)\neq G\left(i\right)\right)$. The four attributes are then calculated by

(7) $$\left\{\begin{array}{c} TP=D*G\\ TN=(1-D)*(1-G)\\ FP=D*(1-G)\\ FN=(1-D)*G \end{array}\right.$$

Then, the precision and recall were calculated by

(8) $$\left\{\begin{array}{c} Precision=\;\frac{TP}{TP+FP}\\ Recall=\;\frac{TP}{TP+FN} \end{array}\right.$$

We also used the weighted F-measure $\left(F_{\beta }^{\omega }\right)$ [47], S-measure $\left(S_{\alpha }\right)$ [48] and E-measure $\left(E_{\xi }\right)$ [49] to evaluate the model performances, similar to [43]. These three metrics are selected to evaluate the precision and recall of the model during testing, as well as to assess the model’s performance. The formulas for these three metrics are as follows.

The weighted F-measure can be calculated by

(9) $$F_{\beta }^{\omega }=\frac{\left(1+\beta^{2}\right){Precision}^{\omega }\cdot {Recall}^{\omega }}{\beta^{2}\cdot {Precision}^{\omega }+{Recall}^{\omega }}$$

where ω represents weight, and β is the adjustable coefficient. In this study, we took ω=1,β=1/2. It is worth noting that the weighted F-measure is a robust metric because it incorporates both precision and recall. The S-measure is calculated by

(10) $$\left\{\begin{array}{c} S=\alpha \cdot S_{0}+\left(1-\alpha \right)\cdot S_{r}\\ S_{0}=\mu \cdot S_{FG}+\left(1-\mu \right)\cdot S_{BG}\\ S_{FG}=\frac{2{\bar{x}}_{FG}}{{\left({\bar{x}}_{FG}\right)}^{2}+1+2\lambda \cdot \sigma_{x_{FG}}}\\ S_{BG}=\frac{2{\bar{x}}_{BG}}{{\left({\bar{x}}_{BG}\right)}^{2}+1+2\lambda \cdot \sigma_{x_{BG}}} \end{array}\right.$$

where FG stands for foreground and BG stands for background. We set α=0.5,μ=0.5,λ=1. $x_{FG}$ represents the probability of the foreground region in the predicted result and the ground truth. $x_{BG}$ represents the probability of the background region in the predicted result and the ground truth. The S-measure will be used to assess the structural similarity of targets in the region.

The E-measure is calculated by

(11) $$\left\{\begin{array}{c} E_{\xi }=\frac{1}{w\cdot h}\sum_{x=1}^{w}\sum_{y=1}^{h}\frac{2\varphi_{GT}\cdot \varphi_{FM}}{\varphi_{GT}\cdot \varphi_{GT}+\varphi_{FM}\cdot \varphi_{FM}}\\ \varphi_{I}=I-\mu_{I}\cdot A \end{array}\right.$$

where *I* is the input binary foreground map. $\mu_{I}$ is the global mean of *I*. *A* is a matrix where all elements have a value of 1, and its size is to the same as that of matric *I*. The E-measure was used to evaluate the performance of the model at the pixel level. We took the mean value of E-measure.

### 4.4. Comparison of the Performance

The results of the comparison are shown in Table 1, where “Null” means that the corresponding results are not provided in the literature. The best performance in each column of the table is highlighted in bold. The data of other existing models were obtained from the corresponding literature and their implementation was carried out using their published code.

From the results on the five different datasets (Table 1, Table 2, Table 3, Table 4 and Table 5, it can be found that the proposed MugenNet shows execellent performance in the CVC-ColonDB and ETIS polyp segmentation datasets (Table 2 and Table 3), showing the best overall performance across the three metrics. It is worth mentioning that our training datasets are sourced from Kvasir and CVC-ClinicDB (Table 4 and Table 5), indicating that our model has good generalization ability and is suitable for the predicting segmentation results on unknown datasets. Although the results on the other three datasets indicate that our model has not achieved the best performance, it is not far from PraNet (only short by 1.6%), which is the current SOTA (the state of the art) performance. We compared the inference time of the different models in Table 6. The results show that with the same training dataset, our model has reduced the inference time by 12% compared with PraNet. At the same time, our model significantly outperforms traditional biomedical image semantic segmentation model (such as U-Net); especially on the ETIS dataset, our model’s performance improvement reaches as high as 13.7% compared with the PraNet model.

The poor performance of the models such as SFA and U-Net on the ETIS dataset suggests that they have a weak generalization ability on unknown datasets. In contrast, our model outperforms the traditional biomedical image segmentation models.

**Figure 3:**
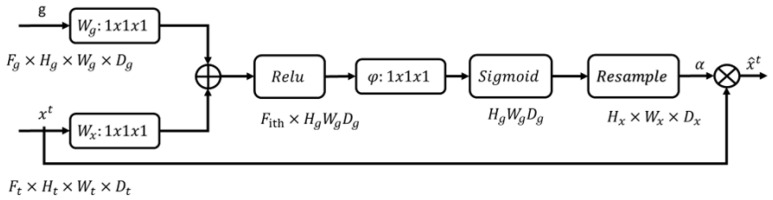
The architecture of the attention gate.

Figure 3 displays the polyp segmentation results of MugenNet on the Kvasir dataset, comparing our model with U-Net, U-Net++, SFA and PraNet, where GT represents the ground truth. Our model can accurately locate polyps of different sizes and textures, then generate semantic segmentation maps. It is worth noting that the results of other models are compared with the results from [43].

**Figure 4:**
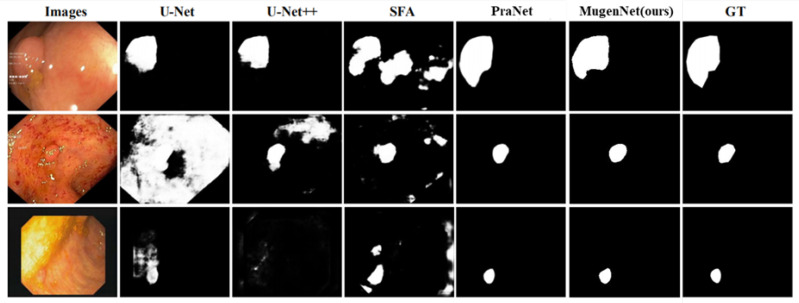
A comparison of the MugenNet with the other four nets (U-Net, U-net++, SFA, Pranet) on the Kvasir dataset.

In addition to the comparison with the four existing models—U-Net, U-Net++, SFA, PraNet in Figure 3—on the Kvasir dataset, we also tested our model on the other four datasets (CVC-300, CVC-ClinicDB, CVC-ColonDB, ETIS-LaribPolypDB,). The results are shown in Figure 4. From Figure 4, our model (MugenNet) can accurately locate the position and size of polyps on the five different datasets. The results show that our model possesses stable and good generalization ability.

**Figure 5:**
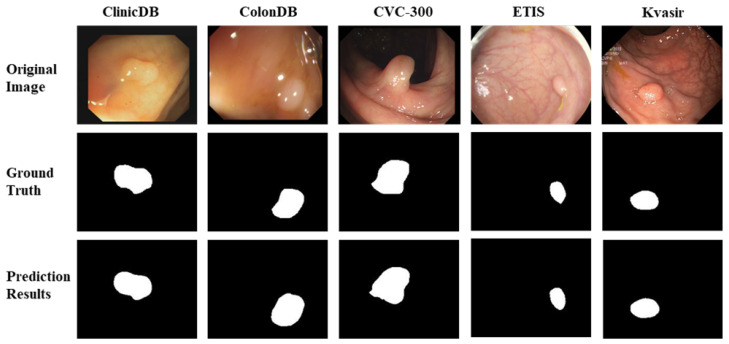
A comparison of the performance of MugenNet on the five datasets (ClinicDB, ColonDB, CVC 300, ETIS, Kvasir).

We also trained our model (MugenNet) on the datasets of CVC-300, CVC-ClinicDB, CVC-ColonDB, ETIS-LaribPolypDB, with the results shown in Figure 5. The examples of the datasets can be found in Figure 5 under original image and ground truth. It can be seen from Figure 5 that our model performs well when trained on the other four polyp datasets. This shows that our model has good robustness (because its performance is insensitive to the training datasets) and can quickly adapt to multiple different datasets.

From the pre-training weight of the DeiT distillation neural network, as shown in Table 6, our model can converge at less than 30 epochs, and the training time is only about 15 min when the batch size is set to 16. LR represents Learning Rate, FPS represents Frames Per Second. Our model’s real-time running speed is approximately 56 frames per second (FPS), which means that our model can be used to process video streaming data during colonoscopy examinations to perform real-time polyp image segmentation tasks.

**Figure 6:**
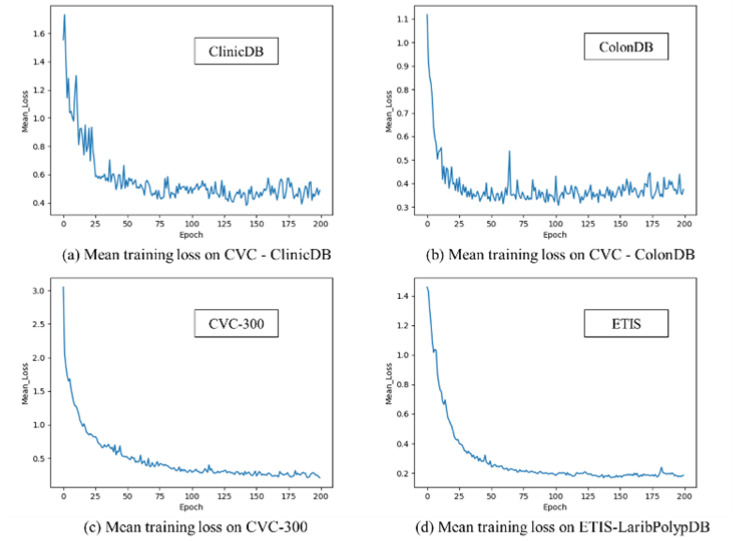
Training process on four datasets.

We tested the Frames Per Second (FPS) of ten models on the training dataset to evaluate the inference speed of the models, as shown in Figure 6. Since the FPS of U-Net and U-Net++ are both below 10 (as shown in Table 6), their inference speed is too slow; hence, they are not shown in Figure 6. It can be observed from Figure 6 that the average FPS of our model reaches 56. It is noted that when FPS is greater than 30, the model was considered reasonable for video streaming data [43]. It can be seen from Figure 6 and Table 6 that our model (MugenNet) has advantages in processing video stream data for the colonic polyp image segmentation.

The comparative results indicate that our model performs well on three unseen datasets, far surpassing traditional CNN-based biomedical image segmentation models such as U-Net, U-Net++, SFA and ResUNet++, and even slightly outperforming PraNet on certain datasets.

**Figure 7:**
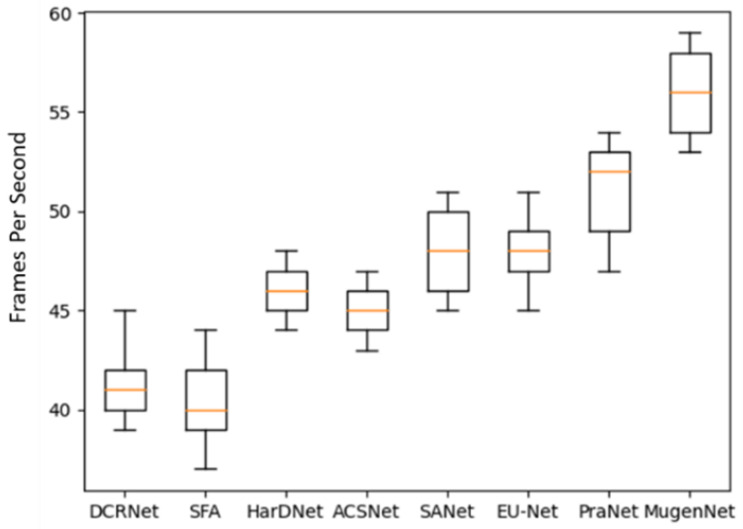
Statistical test on eight models.

It is worth mentioning that the models we compared are all based on convolutional neural networks, while our model (MugenNet) combines Transformer and CNN for colon polyp image segmentation. The experiment results show that the incorporation of Transformer can effectively improve the performance of neural networks, achieving a faster processing speed. In essence, our model (MugenNet) possesses both the global learning capability of Transformer for capturing overall information and the local learning capability of CNN for focusing on specific regions. Therefore, our model achieves an excellent performance in the colon polyp image segmentation. The statistical test on eight models are shown in Figure 7.

### 4.5. Ablation Studies

In this section, five sets of ablation experiments were conducted to evaluate the effectiveness of each component of MugenNet on two different datasets. The results are shown in Table 7, where TB represents the Transformer branch, CB represents the CNN branch, and MM represents the Mugen module.

We used three indicators to evaluate the models, i.e., mDice, mIoU and $F_{\beta }^{\omega }$. Among them, mDice and mIoU were used to evaluate the accuracy of the models, while $F_{\beta }^{\omega }$ was used to evaluate the precision and tecall of the models.

We removed the Transformer branch, the CNN branch, and the Mugen module from MugenNet, respectively, to examine their performances. The result shows that our model (MugenNet) significantly improves the accuracy of semantic segmentation. Compared with the model without the Transformer branch, our model performs better on the ColonDB dataset than the other two cases. The result shows that mIoU increased from 0.591 to 0.678, and mDice increased from 0.667 to 0.758.

The results in Table 7 show that our model can segment colonic polyp image accurately. Our model outperforms the other two models in the ablation study, where either the CNN or the Transformer was omitted. Compared with the backbone neural network, the performance of our model in mIoU improved about 43.34% on the tested dataset (CVC-ColonDB). The results prove the superiority of our model (MugenNet).

## 5. Conclusions

This study proposed a new neural network (MugenNet), which combines Transformer and CNN. MugenNet shows excellent results in performing colonoscopy polyp segmentation tasks. MugenNet was tested on five polyp segmentation datasets (CVC-300, CVC-ClinicDB, CVC-ColonDB, ETIS-LaribPolypDB, Kvasir). Compared with traditional CNNs, our model achieves higher accuracy and faster processing speed. The results of the experiments show that (1) our model can achieve a mean Dice of 0.714 on the most challenging ETIS dataset, specifically, a 13.7% improvement over the current state-of-the-art CNN model (i.e., PraNet), and (2) our model can achieve a better accuracy and faster image processing speed than the traditional CNN, specifically, with the speed of our model being 12% higher than that of PraNet (only taking 30 epochs within 15 min to complete the fitting of the model on the training dataset). Further, the experiments have shown that our model has a good generalization ability and has a robust performance on different datasets, compared with the other models (U-Net, U-Net++, ResUNet++, SFA, PraNet, DCRNet, EU-Net, SANet, ACSNet, HarDNet). Compared with traditional CNNs, our model (MugenNet) achieves faster training speeds. With the use of pre-trained weights from the DeiT distilled neural network, MugenNet converged in just 30 epochs. With a batch size of 16, it only takes 15 min, and the processing speed is about 56 FPS. This performance in processing speed means that our model can be applied to video streaming data processing and real-time clinical polyp segmentation, laying the foundation for the next generation colonoscopy examination techniques.

However, there is still room for improvement. MugenNet is highly dependent on training data, which may impact its performance on previously unseen, complex samples. Additionally, MugenNet’s computational demands are relatively high, which may limit its real-time performance on low-spec hardware, such as in medical endoscopes. Future work will focus on optimizing MugenNet’s architecture and parameters to reduce computational costs and further enhance its generalization capabilities. We also plan to apply MugenNet to other medical imaging segmentation tasks (such as lung infection diagnosis) to validate its applicability and robustness across various fields.

## Figures and Tables

**Table 1:** Comparison results of CVC-300.

Methods	CVC-300 Dataset					
	mDice	mIoU	MAE	$F_{\beta }^{\omega }$	$S_{\alpha }$	$E_{\xi }$
U-Net	0.710	0.627	0.022	0.567	0.793	0.826
U-Net++	0.707	0.624	0.018	0.581	0.796	0.831
ResUNet++	0.763	0.701	0.021	0.613	0.801	0.867
SFA	0.467	0.329	0.065	0.341	0.640	0.644
PraNet	0.871	0.797	0.010	0.843	0.925	0.950
DCRNet	0.856	0.788	0.016	0.830	0.921	0.943
EU-Net	0.837	0.765	0.015	0.805	0.904	0.919
SANet	**0.888**	**0.815**	0.018	0.859	**0.928**	0.962
ACSNet	0.732	0.627	0.016	0.703	0.837	0.871
HarDNet	0.874	0.804	0.019	0.852	0.924	0.948
**MugenNet (Ours)**	0.863	0.795	**0.010**	**0.864**	0.921	**0.972**

**Table 2:** Comparison Results of CVC-ColonDB.

Methods	CVC-ColonDB Dataset					
	mDice	mIoU	MAE	$F_{\beta }^{\omega }$	$S_{\alpha }$	$E_{\xi }$
U-Net	0.512	0.444	0.061	0.498	0.712	0.696
U-Net++	0.483	0.410	0.064	0.467	0.691	0.680
ResUNet++	0.572	0.501	0.058	0.576	0.706	0.701
SFA	0.469	0.347	0.096	0.379	0.634	0.675
PraNet	0.712	0.640	0.043	0.699	0.820	0.847
DCRNet	0.704	0.631	0.052	0.684	0.821	0.840
EU-Net	0.756	**0.681**	0.045	0.730	0.831	0.863
SANet	0.753	0.670	0.043	0.726	0.837	0.869
ACSNet	0.716	0.649	0.039	0.697	0.829	0.839
HarDNet	0.735	0.666	0.038	0.724	0.834	0.834
**MugenNet (Ours)**	**0.758**	0.678	**0.034**	**0.809**	**0.918**	**0.875**

**Table 3:** Comparison results of ETIS.

Methods	ETIS Dataset					
	mDice	mIoU	MAE	$F_{\beta }^{\omega }$	$S_{\alpha }$	$E_{\xi }$
U-Net	0.398	0.335	0.036	0.366	0.684	0.643
U-Net++	0.401	0.344	0.035	0.683	0.683	0.629
ResUNet++	0.423	0.475	0.029	0.628	0.716	0.693
SFA	0.297	0.217	0.109	0.231	0.557	0.531
PraNet	0.628	0.567	0.031	0.600	0.794	0.808
DCRNet	0.556	0.496	0.096	0.506	0.736	0.742
EU-Net	0.687	0.609	0.067	0.636	0.793	0.807
SANet	0.705	0.634	0.025	**0.685**	**0.849**	**0.881**
ACSNet	0.578	0.578	0.059	0.530	0.754	0.737
HarDNet	0.700	0.630	0.025	0.671	0.828	0.854
**MugenNet (Ours)**	**0.714**	**0.636**	**0.019**	0.606	0.677	0.831

**Table 4:** Comparison results of Kvasir.

Methods	Kvasir Dataset					
	mDice	mIoU	MAE	$F_{\beta }^{\omega }$	$S_{\alpha }$	$E_{\xi }$
U-Net	0.818	0.746	0.055	0.794	0.858	0.881
U-Net++	0.821	0.743	0.048	0.808	0.862	0.886
ResUNet++	0.813	0.793	0.053	0.831	0.873	0.878
SFA	0.723	0.611	0.075	0.670	0.782	0.834
PraNet	0.898	0.840	0.030	0.885	0.915	0.944
DCRNet	0.886	0.825	0.035	0.868	0.911	0.933
EU-Net	**0.908**	**0.854**	0.038	0.893	0.917	0.951
SANet	0.904	0.847	0.036	0.892	0.915	0.949
ACSNet	0.898	0.838	0.032	0.882	0.920	0.941
HarDNet	0.897	0.839	0.038	0.885	0.912	0.942
**MugenNet (Ours)**	0.888	0.828	**0.030**	**0.928**	**0.939**	**0.947**

**Table 5:** Comparison results of CVC-ClinicDB.

Methods	CVC-ClinicDB Dataset					
	mDice	mIoU	MAE	$F_{\beta }^{\omega }$	$S_{\alpha }$	$E_{\xi }$
U-Net	0.823	0.755	0.019	0.811	0.889	0.913
U-Net++	0.794	0.729	0.022	0.785	0.873	0.891
ResUNet++	0.796	0.732	0.036	0.803	0.860	0.878
SFA	0.700	0.607	0.042	0.647	0.793	0.840
PraNet	0.899	0.849	**0.009**	0.896	0.936	0.963
DCRNet	0.896	0.844	0.010	0.890	0.933	0.964
EU-Net	0.892	0.846	0.011	0.891	0.936	0.959
SANet	**0.916**	0.859	0.012	0.909	0.939	**0.971**
ACSNet	0.882	0.826	0.011	0.873	0.927	0.947
HarDNet	0.909	**0.864**	0.018	0.907	0.938	0.961
**MugenNet (Ours)**	0.884	0.823	0.017	**0.947**	**0.946**	0.969

**Table 6:** Comparison of the running speed of different models.

Methods	Epoch	LR	Time	FPS	mDice
U-Net	30	$3\times 10^{-4}$	∼40 min	∼8 FPS	0.823
U-Net++	30	$3\times 10^{-4}$	∼45 min	∼7 FPS	0.794
SFA	500	$1\times 10^{-2}$	>60 min	∼40 FPS	0.700
PraNet	20	$1\times 10^{-4}$	∼30 min	∼50 FPS	0.899
**MugenNet**	30	$1\times 10^{-4}$	∼15 min	∼56 FPS	0.875

**Table 7:** Ablation studies of mugennet.

Settings	CVC-ColonDB			ETIS-LaribPolypDB		
	mDice	mIoU	$F_{\beta }^{\omega }$	mDice	mIoU	$F_{\beta }^{\omega }$
Backbone	0.519	0.473	0.637	0.424	0.396	0.381
Backbone + TB	0.726	0.616	0.742	0.649	0.605	0.578
Backbone + CB	0.667	0.591	0.680	0.621	0.563	0.497
Complete Model	**0.758**	**0.678**	**0.809**	**0.714**	**0.636**	**0.606**

## Data Availability

The raw data supporting the conclusions of this article will be made available by the authors on request.
